# Visualization of nerve fibers around the carotid bifurcation with use of a 9.4 Tesla microscopic magnetic resonance diffusion tensor imaging with tractography

**DOI:** 10.1002/hed.25318

**Published:** 2018-06-26

**Authors:** Shin Saito, Hiroyuki Ozawa, Masato Fujioka, Keigo Hikishima, Junichi Hata, Sho Kurihara, Hirotaka James Okano, Kaoru Ogawa

**Affiliations:** ^1^ Department of Otolaryngology ‐ Head and Neck Surgery Keio University School of Medicine Tokyo Japan; ^2^ Okinawa Institute of Science and Technology Tokyo Japan; ^3^ Department of Physiology Keio University School of Medicine Tokyo Japan; ^4^ Department of Otorhinolaryngology Keio University School of Medicine Tokyo Japan; ^5^ Division of Regenerative Medicine the Jikei University School of Medicine Tokyo Japan

**Keywords:** head and neck, MR diffusion tensor imaging, MR diffusion tensor tractography, postmortem study, ultra‐high magnetic field MR

## Abstract

**Background:**

Precise imaging of nerves have been challenging in the head and neck region, mainly due to low spatial resolution. Here, we investigated how nerves in the head and neck region could be visualized using an ultra‐high magnetic field MR system.

**Methods:**

We used formol‐carbol‐fixed human cadaveric necks and obtained MR diffusion tensor images (DTIs) using a 9.4 Tesla (T) ultra‐high magnetic field MR system. Afterward, we prepared tissue sections and checked the anatomic relationships between the neurons and the carotid artery in order to confirm that the visualized fibers are indeed neuron fibers.

**Results:**

We were able to identify nerves, including the vagus nerve, the hypoglossal nerve, and the spinal‐accessory nerve. Hematoxylin‐eosin stained histological sections confirmed neuron fibers in the same anatomic position.

**Conclusion:**

This technique has the feasibility to be applied for a more accurate anatomic understanding, maybe even close to a histological level.

## 1 INTRODUCTION

In head and neck surgery, identifying and preserving nerves is essential, because loss of function of those nerves running deep in the neck will directly lead to various symptoms, such as dysphagia, hoarseness, and anarthria, deteriorating the patient's quality of life. However, detecting the nerves during the operation is sometimes difficult mainly due to a couple of reasons. First, the anatomy of the nerves varies among individuals. For example, various anatomies of the vagus nerves^1^ and spinal accessory nerves^2^ have been reported. Second, nerves can be displaced by surrounding structures (such as tumors or swollen lymph nodes caused by cancer metastasis), leading to an “abnormal anatomy.” Furthermore, these masses can sometimes invade the nerves. Unfortunately, at present, there is no definite method to judge whether preservation of the nerves is possible until we open the surgical field. Visualization of the courses of nerves by imaging enables us to obtain a more accurate preoperative simulation, helping us carry out surgery in a more precise and safer way.

Recently, techniques called diffusion tensor imaging (DTI) and diffusion tensor tractography (DTT) have been reported to be useful in visualizing both central and peripheral nerve fibers.^3^, ^4^, ^5^, ^6^, ^7^, ^8^, ^9^, ^10^, ^11^, ^12^, ^13^, ^14^, ^15^, ^16^, ^17^ The DTI technique is an MR acquisition modality that measures the diffusion anisotropy of water molecules. Because water molecules tend to diffuse along axons, neurofilaments, and nerve sheaths, DTI makes it possible to delineate the orientation of nerve fibers. The DTT technique refers to the analysis and reconstruction of the data obtained by DTI, by which the orientation of nerve fibers can be followed to trace specific neural pathways, such as that of the corticospinal tract in the brain, the spinal cord, and peripheral nerves. However, due to lack of spatial resolution, few reports have been published to be able to use this technique in the head and neck region at a clinically useful level.^8^, ^9^, ^10^ A higher resolution imaging is required, and an ultra‐high magnetic field MR system has chances to overcome this problem, however, it is unsure how nerves in this area would be imaged using this technique. By using postmortem specimens, we attempted to visualize nerve fibers focusing on the area surrounding the carotid bifurcation, where multiple cranial and peripheral nerves run in a complex way. Here, we report how nerve fibers are made visible in the head and neck region when MR imaging and tractography are performed using ultra‐high magnetic field systems.

## 2 MATERIALS AND METHODS

### 2.1 MRI equipment

The 9.4T Biospec 94/20 MRI has a transmitter and receiver coil, a conventional birdcage circuit, and an inner diameter of 72 mm (Bruker BioSpin, Ettlingen, Germany).

### 2.2 High resolution imaging

Diffusion tensor imaging (DTI) data was acquired using a 3D spin‐echo sequence based on a Stejskal‐Tanner diffusion preparation. The imaging parameters were: Repetition Time (TR)/Echo Time (TE)/δ/Δ = 500/22.5/4/10 ms, 1500 s/mm^2^, motion probing gradients = 30 axes, b0 = 2 volumes, and isotropic resolution at 250 um. First, 2 samples were taken together with the scan time of 68 hours 32 minutes, and the next 3 samples were with 80 hours 50 minutes.

### 2.3 Micro level fiber tract map

We used the Diffusion Toolkit and TrackVis^11^ (Massachusetts General Hospital, Boston, MA) for DTI and DTT analysis. We set a length threshold of over 20 mm to exclude short fibers and also set multiple regions of interest to include or exclude visualized fibers.

### 2.4 Three‐dimensional visualization

We used Amira (Mercury Computer Systems, Chelmsford, MA) for 3D visualization. We overlapped a CT image of the artery, which was obtained from the LaTheta LCT‐200 in vivo micro‐CT scanner before the MRI scan, with the MRI data so that the anatomic position relationship was easier to determine.

### 2.5 Samples

**Figure 1:**
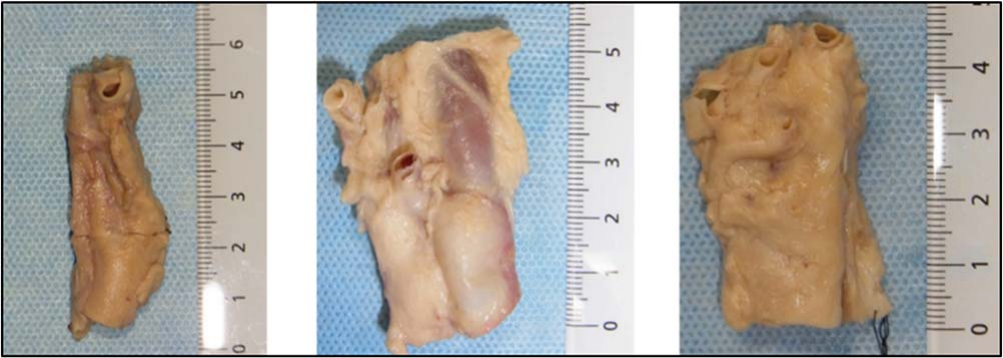
Pictures of the representative specimens. Measures are in centimeters. The top is the cranial side [Color figure can be viewed at http://wileyonlinelibrary.com]

Five sides of the necks were dissected from 3 formol‐carbol fixed human cadaveric bodies (range 84‐96 years old). The causes of death were pneumonia, colon cancer, and unknown reason, respectively. None had scars or operation history to the neck. The necks were carefully dissected so that the tissues surrounding the carotid sheath were taken out as a block (Figure 1).

The tissues were kept in phosphate‐buffered saline containing 1‐mM Gadopentetate dimeglumine (Magnevist; Bayer Healthcare Pharmaceuticals, Wayne, NJ) and 0.5% azide for 2 weeks and then replaced in Fluorinert (Sumitomo 3M Limited, Tokyo, Japan) when taking the images.

After taking MRI, we made paraffin‐embedded cross sections from the specimens and stained them with hematoxylin‐eosin and S‐100, and compared them with the axial view of the MRI.

The MRI acquisition was performed by K.H. and J.H., data analysis of the MRI was performed by S.S., and histology preparation was performed by K.S. Total analysis of the obtained data was performed by Ear, Nose, and Throat surgeons, S.S., H.O., and F.M.

## 3 RESULTS

**Figure 2:**
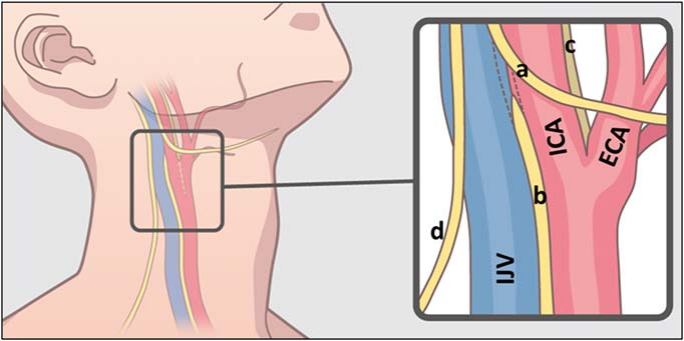
Scheme of the anatomy of the human neck. Black frame represents the approximate area dissected in this study. Blue structure is internal jugular vein (IJV), red is carotid artery (ICA: internal carotid artery, ECA: external carotid artery). Yellow lines represent the nerves. (a: hypoglossal nerve, b: vagus nerve, c: glossopharyngeal nerve, d: spinal accessory nerve) [Color figure can be viewed at http://wileyonlinelibrary.com]

**Figure 3:**
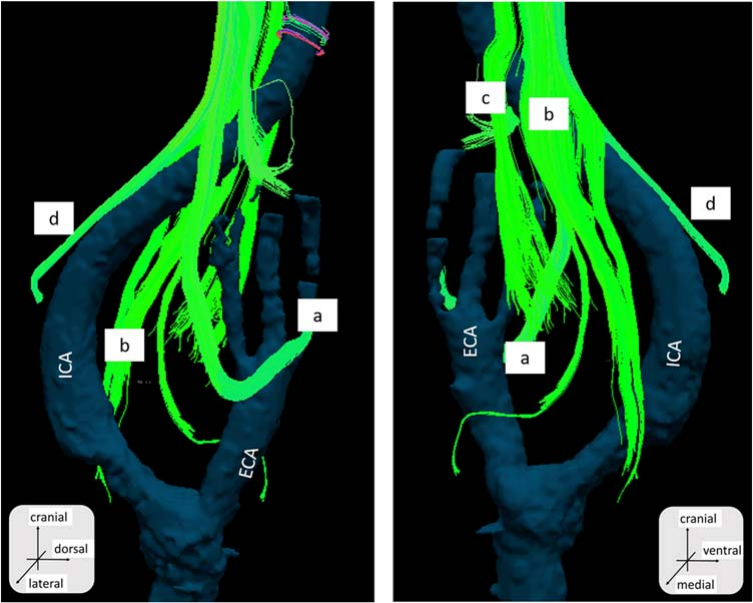
The diffusion tensor tractography fusion with artery structure obtained from CT. Right side neck. Sample number 1. a, The hypoglossal nerve; b, the vagus nerve; c, the glossopharyngeal nerve; and d, the spinal accessory nerve. ECA, external carotid artery; ICA, internal carotid artery [Color figure can be viewed at http://wileyonlinelibrary.com]

**Figure 4:**
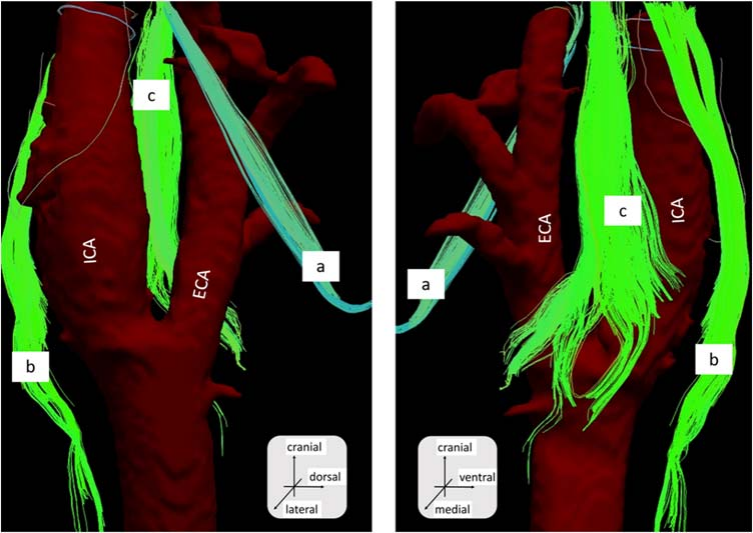
The diffusion tensor tractography fusion with artery structure obtained from CT. Right side neck. Sample number 2. a, The hypoglossal nerve; b, the vagus nerve; and c, the glossopharyngeal nerve. ECA, external carotid artery; ICA, internal carotid artery [Color figure can be viewed at http://wileyonlinelibrary.com]

The images shown in this article are representative of those obtained from the necks of the cadaveric bodies (n = 5). We were able to identify fibers, whose anatomic relationships with the internal and external artery were compatible with the hypoglossal nerve, the spinal accessory nerve, and the vagus nerve (Figures 2, 3, and 4).

The hypoglossal nerve was relatively easy to detect, due to its unique running course. The fibers were found to run cranial to caudal, diagonally running anteriorly and crossing the external carotid artery, and then turning in a cranial‐anterior direction. The spinal accessory nerve was also determined by its unique tract descending anterior to posterior. The vagus nerve was identified by its course running medial‐posterior to the common carotid artery. The glossopharyngeal nerve was detected by the bundle of fibers running medial to the carotid artery between the internal and external carotid arteries. Numerous fibers branching from the glossopharyngeal nerve ended at the carotid bifurcation (Figures 3 and 4). The nerves that we were able to detect slightly varied among samples, which we think was due to technical variation during dissection.

**Figure 5:**
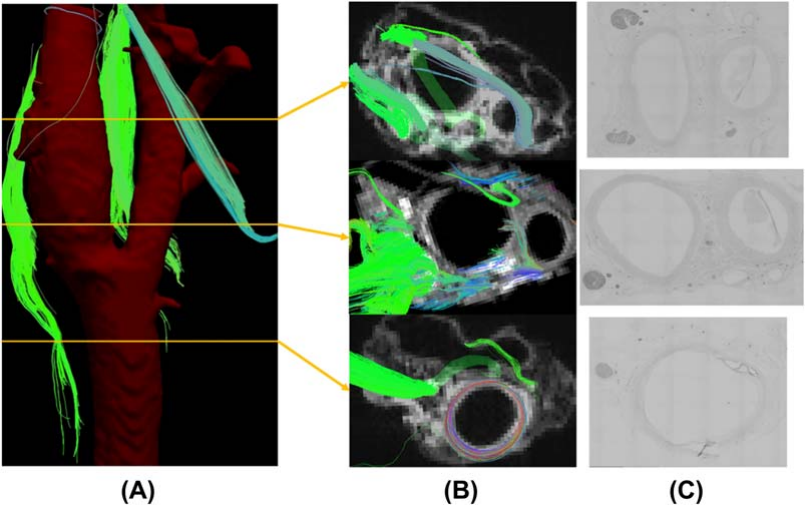
A, The approximate level of the axial slices. B, The axial reconstruction of the MRI revealed multiple fibers running near the carotid sheath. C, Immunostaining of S‐100 confirmed that the fibers were compatible with nerves [Color figure can be viewed at http://wileyonlinelibrary.com]

Hematoxylin‐eosin and immunostaining revealed the anatomic relationships of the vessels and the neuron fibers, which were very similar to those that we obtained from the axial view of the MRI (Figure 5).

**Figure 6:**
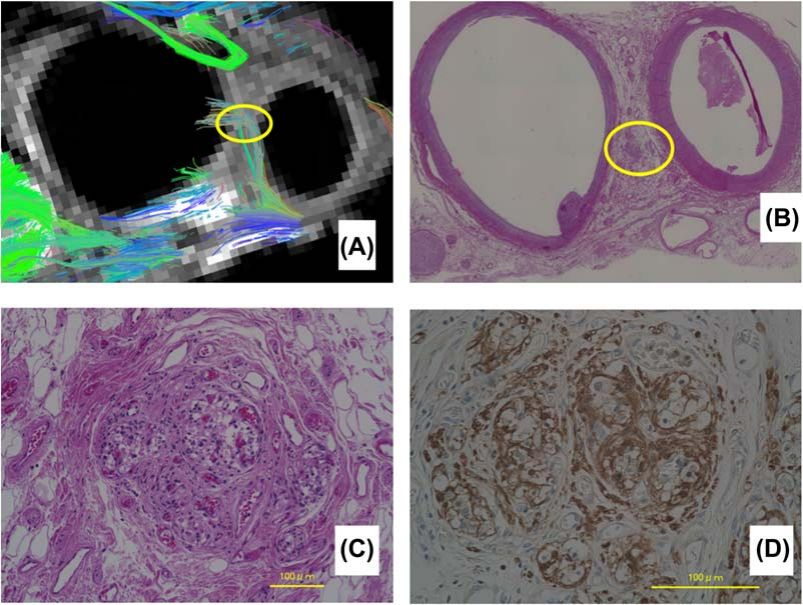
A, The axial slice of the MRI at the level near the bifurcation. Multiple fibers are seen to run towards the bifurcation (yellow circle). B, Hematoxylin‐eosin staining of the same specimen with a low power field. C, Hematoxylin‐eosin staining. The high power field of the yellow circle in B showing structures similar to the carotid body. D, The S‐100 immunostaining. High power field. Same slice of figure C [Color figure can be viewed at http://wileyonlinelibrary.com]

We were also able to detect fibers running medial to lateral, toward and terminating at the carotid bifurcation. From the sections near the same level of the MRI, we were able to determine a microstructure between the arteries, which was compatible with the carotid body (Figure 6).

These data indicated that the fibers leading toward the carotid bifurcation were consistent with the nerves innervating the carotid body.

## 4 DISCUSSION

In recent years, DTI, an MRI technique that allows us to delineate the axonal organizations, has been reported to be useful in understanding nerve anatomy^3^, ^12^, ^13^, ^14^, ^15^, ^16^, ^17^ and nerve disorders.^4^, ^5^, ^6^, ^7^, ^18^ In the head and neck region, reports are limited. Akter et al^8^ reported that it was possible to predict the anatomic relationship of the nerves and tumors in 4 of 5 patients with head and neck tumors by using this method in a 3T MR system. Gallagher et al^9^ reported that this technique was useful in preoperative workup, applying this image to a patient with C4 nerve schwannoma. Manoliu et al^10^ successfully imaged mandibular nerves in 8 healthy volunteers. However, the images in these reports are still somewhat vague compared to the actual surgical field, which is probably due to lack of spatial resolution. In order to overcome this problem, a higher resolution imaging tool is demanded, which we think has chances to be solved by using a higher magnetic field imaging. However, there has been no previously reported studies on how nerves of the head and neck could be visualized using an ultra‐high MRI. Although still a postmortem study, this is the first study to report visualization of nerve fibers in the head and neck region using a 9.4T magnetic field MRI. We successfully identified the major nerves running close to the carotid artery, such as the hypoglossal nerve, the spinal accessory nerve, and the vagus nerve. We were also able to track fibers leading to the carotid bifurcation where the carotid body is located. These tracked fibers were very similar to those previously reported by Schulz et al^19^ based on macrodissection. Thus, by using this method, tractography has the feasibility to reveal anatomies in a very detailed manner.

Although accumulated data of human anatomy, mainly based on cadaveric dissection, has revealed most of major anatomies in the head and neck area, there are still anatomies yet to be understood. For example, the previously mentioned report of innervation of the carotid body was not well understood until Shultz et al^19^ reported it in 2016. In the same way, by applying this method, we have the chance to clearly understand anatomies, such as the innervation of the tongue, olfactory nerve distribution, postsurgical nerve recovery, etc.

There are a few limitations to apply this technique in clinical practice. One issue is the imaging time. This approach currently takes about 25 to 30 hours to obtain 1 sample. This was mainly due to 2 reasons. First, we chose a conventional 3D spin‐echo sequence instead of echo‐planner imaging, so that we were able to exclude artifacts as much as possible. Second, the high resolution we set (250‐um isotropic resolution) led us to take more time than the previously reported methods.

Another issue is that when the technique is applied to a living body, the signals from the vessels (especially the carotid artery) can make it very difficult to analyze the DTI data. The blood flow and the pulse from the artery can cause many artifacts, which are critical in reconstructing the neurofibers.

These issues, the imaging time and the artifacts, are challenging to solve, and currently restricts the use of this technique to cadaveric situations.

Still, we hope that in the near future, this technique can be applied to clinical practice, which would allow us to understand the location of the nerves and their relationships with the surrounding structures preoperatively, in a very precise manner, maybe even close to a histological level.

## 5 CONCLUSION

This is the first study, to the best of our knowledge, to report visualization of nerve fibers in the head and neck region using a 9.4T ultra‐high magnetic field MRI. By using this technique, tractography has the feasibility to reveal anatomies close to a macrodissection. We think this technique can be applied to get a more accurate anatomic understanding, maybe even close to a histological level.

## ACKNOWLEDGMENTS

The authors thank Hiroki Ohta and Yoshinori Kawai for preparation of the cadaveric samples. This work was supported by a grant from the Japanese government MEXT KAKENHI (24592560, 15H04991, 15K15624, and 17K19732), the Takeda Science Foundation, and a grant by the Jikei University Research Fund and the Strategic Prioritising Research Fund.
